# ‘We don’t know for sure’: discussion of uncertainty concerning multigene panel testing during initial cancer genetic consultations

**DOI:** 10.1007/s10689-019-00154-4

**Published:** 2019-11-26

**Authors:** Niki M. Medendorp, Marij A. Hillen, Pomme E. A. van Maarschalkerweerd, Cora M. Aalfs, Margreet G. E. M. Ausems, Senno Verhoef, Lizet E. van der Kolk, Lieke P. V. Berger, Marijke R. Wevers, Anja Wagner, Barbara A. H. Caanen, Anne M. Stiggelbout, Ellen M. A. Smets

**Affiliations:** 1 Department of Medical Psychology – Amsterdam UMC, University of Amsterdam, P.O. Box 22700, 1100 DE Amsterdam, The Netherlands; 2 Amsterdam Public Health Research Institute, Amsterdam, The Netherlands; 3 Cancer Center Amsterdam, Amsterdam, The Netherlands; 4 Department of Clinical Genetics – Amsterdam UMC, University of Amsterdam, Amsterdam, The Netherlands; 5 Division of Biomedical Genetics, Department of Genetics, University Medical Center Utrecht, Utrecht University, Utrecht, The Netherlands; 6 Department of Clinical Genetics, Leiden University Medical Center, Leiden, The Netherlands; 7 Family Cancer Clinic, Netherlands Cancer Institute, Amsterdam, The Netherlands; 8 Department of Genetics, University Medical Center Groningen, Groningen, The Netherlands; 9 Department of Human Genetics, Radboud University Medical Center, Nijmegen, The Netherlands; 10 Department of Clinical Genetics, Erasmus University Medical Center, Rotterdam, The Netherlands; 11 Department of Clinical Genetics, Maastricht University Medical Center, Maastricht, The Netherlands; 12 Medical Decision Making, Department of Biomedical Data Sciences, Leiden University Medical Center, Leiden, The Netherlands

**Keywords:** Genetic testing, Ambiguous, Information provision, Genetic counselors, Simulated patients

## Abstract

Pre-test counseling about multigene panel testing involves many uncertainties. Ideally, counselees are informed about uncertainties in a way that enables them to make an informed decision about panel testing. It is presently unknown whether and how uncertainty is discussed during initial cancer genetic counseling. We therefore investigated whether and how counselors discuss and address uncertainty, and the extent of shared decision-making (SDM), and explored associations between counselors’ communication and their characteristics in consultations on panel testing for cancer. For this purpose, consultations of counselors discussing a multigene panel with a simulated patient were videotaped. Simulated patients represented a counselee who had had multiple cancer types, according to a script. Before and afterwards, counselors completed a survey. Counselors’ uncertainty expressions, initiating and the framing of expressions, and their verbal responses to scripted uncertainties of the simulated patient were coded by two researchers independently. Coding was done according to a pre-developed coding scheme using The Observer XT software for observational analysis. Additionally, the degree of SDM was assessed by two observers. Correlation and regression analyses were performed to assess associations of communicated uncertainties, responses and the extent of SDM, with counselors’ background characteristics. In total, twenty-nine counselors, including clinical geneticists, genetic counselors, physician assistants-in-training, residents and interns, participated of whom working experience varied between 0 and 25 years. Counselors expressed uncertainties mainly regarding scientific topics (94%) and on their own initiative (95%). Most expressions were framed directly (77%), e.g. *We don’t know*, and were emotionally neutral (59%; without a positive/negative value). Counselors mainly responded to uncertainties of the simulated patient by explicitly referring to the uncertainty (69%), without providing space for further disclosure (66%). More experienced counselors provided less space to further disclose uncertainty (*p* < 0.02), and clinical geneticists scored lower on SDM compared with other types of counselors (*p* < 0.03). Our findings that counselors mainly communicate scientific uncertainties and use space-reducing responses imply that the way counselors address counselees’ personal uncertainties and concerns during initial cancer genetic counseling is suboptimal.

**Electronic supplementary material:**

The online version of this article (doi:10.1007/s10689-019-00154-4) contains supplementary material, which is available to authorized users.

## Introduction

Multigene panels involve sequencing multiple genes simultaneously to identify genetic cancer predispositions [1]. An advantage of panel testing is the increased yield of genetic diagnosis, particularly in families fitting multiple cancer syndromes [2]. However, sequencing a large number of genes increases the level of uncertainty compared with more targeted tests [2, 3], as it increases the possibility to identify uncertain and unsolicited findings. This subsequently generates uncertainties, such as what and how to communicate to counselees during pre-test and post-test counseling [2].

Three categories of medical uncertainties have been identified in the current literature: (i) scientific, (ii) practical, and (iii) personal [4]. Scientific uncertainty comprises uncertainties regarding the evidence on diagnosis and implications for prevention, treatment and prognosis [5]. Practical uncertainty applies to processes of care, e.g. whether to communicate test results without clinical relevance. Lastly, personal uncertainty pertains to psychosocial and existential consequences for patients, e.g. whether uncertain information should be disclosed to relatives [4].

During genetic counseling, large amounts of genetic information are generally provided by counselors to increase counselees’ knowledge and promote their understanding [6]. However, given the increased level of uncertainty concerning panel testing, provision of uncertain information is expected to be more extensive in consultations about this type of test. One study showed that counselors struggle with the amount of uncertain information they need to provide in the context of panel testing, and that they vary in their communication about uncertainty during pre-test counseling [7]. This implies that not all decisions of individual counselees regarding testing are based on the same information and that they may, therefore, strongly depend on the counselor. Ideally, counselees are adequately provided with information, including information about uncertainties, irrespective of their individual counselor [6]. As counselees often engage in genetic counseling hoping to become more certain about their medical situation, e.g., by finding out whether or not they are a carrier, counselors need to make counselees aware of the possible uncertainties associated with panel testing to manage their expectations [8]. In particular, from counselees’ perspective there is no *best* option in deciding about multigene panel testing, since testing may involve both harms and benefits for them. Therefore, disclosing uncertainties that may result from panel testing, is necessary to enable counselees to weigh the pros and cons of undergoing such a test. Therefore, their autonomy is promoted as counselees are allowed to be involved in decision making and to decide together with the counselor according to their personal values, i.e. shared decision making (SDM) [9].

Similar to the content of uncertain information, the *manner* in which counselors communicate uncertainty may also vary. The effect of uncertainty communication may differ depending on its framing, e.g. adding a positive or negative value [10, 11]. One study showed that people’s attitude and behavior towards self-examination differ when adding a positive or negative value to risk information [12]. It is plausible that this also applies to the framing of uncertainty in consultations about panel testing; one may be more inclined to opt for panel testing when uncertainty is communicated in positive terms [13]. In the context of decision making, non-directive counseling is required to enable counselees to decide without being steered by their counselor [14]. However, some may argue that a positive or negative framing helps counselees contemplate their decision in certain situations. For example, when counselees are strongly convinced that genetic testing has a negative outcome, counselors may use framing to help grow more realistic expectations in counselees.

Primarily counselors introduce uncertainties during genetic counseling, but counselees may express uncertainty as well [15]. Counselees’ uncertainties may have existed before counseling, or be evoked by the uncertainties introduced by the counselor (i.e. as a reaction to the provided information). If counselees express uncertainties, counselors need to respond to and deal with these during genetic counseling. Studies have examined how physicians respond to patient expressions like uncertainty [16], and how patients can be supported in dealing with these [17]. However, to our knowledge, no studies have investigated how counselors handle uncertainties expressed by counselees during genetic counseling.

Hence, it is unknown whether and how counselors communicate about and respond to uncertainty resulting from multigene panel testing, and to what extent counselees are involved in deciding about panel testing. Therefore, this study aimed to gain insight into counselors’ discussion of uncertainty by (1) examining *which* uncertainties counselors discuss and *how*, (2) examining how they respond to counselees’ expressions of uncertainty, and (3) describing the extent to which counselors engage in SDM in initial cancer genetic counseling about multigene panel testing with a simulated patient (SP). Moreover, this study attempted to (4) explore associations between counselors’ background characteristics and their communication of uncertainty, responses to uncertainty and the extent of SDM.

## Materials and methods

### Design and ethics

In this cross-sectional observational study, counselors discussed a multigene panel test with a SP. Creating so-called *simulated consultations* eliminates variation at patient level, enabling the use of one instead of multiple consultations per counselor. This method has previously been successfully applied [18]. The Medical Ethics Review Board of the Academic Medical Center approved the study and waived the need for ethico-legal adjudication as it would have no serious impact on participating counselors.

#### Scenario

All simulated consultations were based on the same scenario: a highly-educated male counselee who has had three types of cancer and visits for his first genetic counseling appointment concerning a pretest-counseling session about the option to perform a multigene panel test for cancer. One type of counselee was used in this study to enable standardization across consultations and thus to compare communication between counselors. A male counselee was chosen for practical reasons.

#### Simulated patients (SPs)

Two experienced male actors, comparable in age (± 60 years old), were trained to act as SP1 and SP2 according to a script (Supplement A). The script contained background information, e.g. the reason for seeking cancer genetic counseling, and instructions to provide two statements indicating uncertainty and to ask two specific questions during the consultation, e.g., ‘Oh, what if something unknown is determined... What am I supposed to do then..'. Further, SPs were instructed to follow the lead of the counselor, providing information or asking questions only when prompted.

SPs were trained twice in 2-h sessions to review the script and practice the case with a clinical geneticist in the presence of the research team. After the first session, the SPs acted in four pilot consultations with counselors not participating in this study, to test the script and SPs’ behavior, and to further adjust the script. During the second session, the final script was discussed and practiced.

### Participants

In the Netherlands, genetic testing for cancer is performed at seven university medical centers and one oncology-specific tertiary referral center. Eligible for participation in the present study were all counselors (i.e., clinical geneticists, residents and interns, physician assistants (in training) and genetic counselors) affiliated with these centers and performing cancer genetic counseling. The study was advertised at all centers, and interested counselors received more details from the first author. To create a large and heterogeneous sample, clinical geneticists (both staff and residents), physician assistants and genetic counselors varying in years of working experience were recruited. To create an equal distribution across the centers, the aim was to recruit at least five counselors per institute, three of which were clinical geneticists.

### Procedure

Data were collected between September 2017 and March 2018. When counselors agreed to participate, a consultation with a SP was scheduled at the counselors’ own institute. Three weeks before this consultation, counselors gave written informed consent and completed a questionnaire assessing their background characteristics (T0). One week before the consultation, they received a brief instruction letter, a simulated medical file, and the SP’s pedigree. Counselors were instructed to conduct their consultation as they would do in routine clinical care and to take the time commonly needed for this type of consultation, which was on forehand specified to vary between 30 and 60 minutes in standard practice. Consultations were video-recorded and counselors completed a questionnaire assessing their perception of realism and degree of SDM during this consultation (T1) immediately afterwards.

### Measures

#### Background characteristics (at T0)

The following background characteristics of counselors were assessed:

- Socio-demographic, i.e. age and gender, and practice characteristics, i.e. professional training, years of counseling experience, and experience in communication training.
- Confidence in discussing uncertainty, using a 5-item questionnaire which was developed for this study based on previous literature and existing questionnaire items from measures assessing related constructs [19]. All items were answered on a 7-point Likert response scale (1 = completely disagree and 7 = completely agree), with a maximum total score of 35. An example of one item is: *I am very capable in discussing uncertainty about a panel test*.
- Uncertainty tolerance, using the 5-item ‘Anxiety because of uncertainty’-scale of the Physicians’ Reaction to Uncertainty (PRU) questionnaire with a 6-point Likert response scale, with a maximum total score of 30 [20]. This questionnaire was translated to Dutch using forward–backward translation [21].
- Attitude towards SDM, using a 4-item questionnaire with a 7-point Likert response scale [22]. Maximum total score was 24 and scores < 12 were classified as a positive attitude and scores ≥ 12 as negative. Items were translated to Dutch using forward–backward translation [21].
- Perception of colleagues’ attitude towards SDM (i.e. perceived social norm) using a 2-item questionnaire which was developed based on previous literature [23]. Answers were given on a 7-point Likert response scale (1 = completely disagree and 7 = completely agree; maximum total score of 14). For example, one item is *Most counselors working in my center think it is important to apply shared decision making in consultations about panel tests*.

#### SDM (at T1)

We assessed counselors’ perception of the degree of SDM during the simulated consultation, using the Dutch version of the 9-item SDM-Q-Doc with a 6-point Likert response scale [24]. This questionnaire has been shown to have a good acceptance and reliability.

#### Realism (at T1)

Counselors’ perceived realism of the consultation was measured using a 3-item questionnaire with a 7-point Likert response scale (1 = completely disagree and 7 = completely agree). We used an adapted version of a questionnaire with a Cronbach’s alpha = 0.84, that has been used in previous studies of our research group (for example [25]). In addition, realism of SPs’ behavior was measured using a 2-item questionnaire (Cronbach’s alpha = 0.79) with a 7-point Likert response scale (1 = not at all and 7 = totally).

#### Content coding of consultations

##### Coding of counselors’ expressions of uncertainty

All consultations were coded by two coders (NM and PvM) independently. To identify counselors’ expressions of uncertainty and their responses to counselees’ utterances of uncertainty, videos were coded according to a coding scheme using The Observer XT software for observational analysis of video recordings [26]. Details of the development and content of the coding scheme are provided in Supplement B. The final coding scheme included 42 detailed codes covering 13 topics about which counselors could express uncertainty, categorized among four issues: scientific test-related, scientific disease-related, practical and personal uncertainties [4]. For each expression we also coded its *initiation*, i.e., counselor initiated or stimulated by the counselee, and *framing*, i.e., whether uncertainty was expressed directly, i.e. a decisive expression; e.g. *Wedon’t knowwhat it means*, or indirectly, i.e. a hesitant expression; e.g. *This pathogenic variant isprobablynot related to your cancer*. Moreover, we coded whether neutral, positive or negative terms were used when expressing uncertainty, i.e. respectively ‘only’ disclosing uncertainty, adding a positive value (e.g. uncertainty does not necessarily imply something detrimental) or adding a negative value (e.g. an uncertain finding may turn out to be a pathogenic variant) to emphasize either one of the implications of uncertainty.

##### Coding of counselors’ responses to counselee expressions of uncertainty

To code counselors’ responses to SPs’ utterances of uncertainty, the Verona Coding Definitions of Emotional Sequences (VR-CoDES) were used [16]. This system allows coding counselors’ responses to SPs’ scripted and spontaneous utterances of uncertainty in terms of their (a) explicitness, and (b) space. An *explicit* response includes a clear reference to counselees’ uncertainty whereas a *non-explicit* response does not. Space refers to whether counselors reduced or provided space for the counselee to further disclosure uncertainty. An example of a response that reduces space is when a counselor switches to another subject, e.g. SP: *I’m worried about whether my children will develop cancer;* Response of counselor: *Does any of your children know that you’re here today?*. Providing space is subdivided into *content* space, i.e., to explore the content of the uncertainty, for example; *What is the reason you want to know whether you carry a pathogenic variant or not?*, and *affective* space, i.e., to explore the affect associated with the uncertainty, for example; *Why do you think you’ll have difficulty in dealing with an uncertain variant?*. The combination of these characteristics results in five response categories: (1) non-explicit, reducing space; (2) non-explicit, providing space; (3) explicit, reducing space; (4) explicit, providing *content* space; and (5) explicit, providing *affective* space (categories of VR-CoDES responses are presented in Fig. 1 in Supplement C).

##### Coding of SDM and realism

The two coders independently assessed the degree of SDM, using the 9-item SDM-Q with a 6-point Likert response scale [27], adapted to be used as a coding instrument, and realism of SPs’ behavior, using a self-developed 3-item coding instrument with a maximum score of 6 per item.

### Interrater reliability

After the two coders coded ten observations, interrater reliability of coded uncertainties, responses to uncertainty, SDM and realism was calculated. Since the interrater reliability of coded uncertainties and responses was sub-optimal (mean κ = 0.16; range 0.09–0.28), it was decided to double-code all observations independently. This would enable discussing and reaching consensus on any coding discrepancies, and thereby increase reliability. Reliability analyses on SDM data showed a moderate mean κ = 0.38; range 0.03–0.92. Reliability of realism was judged to be *substantial* (Cohen’s Kappa κ = 0.60). After every five observations, coders met to compare their coding and if they disagreed, consensus was reached through discussion.

### Statistical analysis

All statistical tests were performed using SPSS Statistics, version 21. Data distributions were checked for normality using visual inspections combined with parametric tests. Descriptive statistics were used to summarize counselors’ background characteristics, i.e. level of experience, professional training, uncertainty tolerance, confidence in communicating uncertainty, attitude towards SDM and perceived social norm about SDM. As a manipulation check, differences between consultations of the two SPs (i.e., differences in type of counselor, work experience, received communication training and mean duration of consultation) were assessed using independent *t* test or χ^2^-test, whichever was deemed more appropriate. Further, a consensus score was calculated of how realistic SPs’ behavior was rated by the two observers. Differences in realism scores as rated by counselors as well as observers were assessed using independent *t * test or χ^2^-test.

Counselors’ expressed uncertainties, its initiation and framing, and the responses to uncertainties were summarized using descriptive statistics. To determine whether expressions of the four uncertainty issues (i.e. scientific test-related, scientific disease-related, practical and personal) differed in how they were framed, ratios of framing were calculated by dividing all uncertainty expressions related to one issue with one type of framing (e.g. all scientific test-related uncertainties framed in direct positive terms) by the total number of uncertainty expressions related to that particular issue. Subsequently, a χ^2^-test was performed to test for differences in ratios. Further, a consensus score of the ratings of SDM by the two observers was calculated after which differences between observers’ and counselors’ ratings of SDM were assessed using independent t-test or χ^2^-test. This consensus score of SDM was used in further analyses on SDM.

The associations between the frequency of the four uncertainty issues and counselors’ background characteristics were assessed using Pearson’s correlation or regression analyses. This was also done to determine whether counselors’ background characteristics were correlated with each other. Further, we examined whether counselors’ responses to uncertainties, e.g. providing space, and counselors’ SDM scores were correlated with counselors’ background characteristics using Pearson’s correlation or regression analyses. Moreover, associations between the frequency of uncertainty issues and counselors’ SDM scores, and between counselors’ responses to uncertainties, e.g. space providing, and counselors’ SDM scores were assessed using similar analyses.

Finally, a post-hoc power analysis was performed using G*Power version 3.1.9.2 [28]. Using an alpha of 0.05, we had a 50% power to determine medium effects (effect size of 0.3) with our sample size of 29. For all analyses, a significant level of *p* < .0.05 was used.

## Results

### Sample characteristics

A total of 29 counselors from all Dutch genetic centers participated; range 1–6 per center. Characteristics of participants are shown in Table 1. Mean duration of the consultation was 34 min (SD = 7.1; range 20.4–47.5). Of all counselors, 16 had a consultation with SP1 and 13 saw SP2; there were no differences in counselors’ characteristics between those who saw SP1 or SP2.

**Table 1:** Counselors’ characteristics (N = 29)

	n (%)	M ± SD (range)
Age (in years)		43.5 ± 1.9 (26–63)
Female	23 (79.3)	
Professional training		
Clinical geneticist	16 (55.2)	
Genetic counselor	5 (17.2)	
Physician assistant-in-training	5 (17.2)	
Resident	1 (3.4)	
Intern	2 (6.9)	
Work experience (in years)		11.8 ± 1.54 (0–25)
Received training in communication		
Never	3 (10.3)	
1–2 times	11 (37.9)	
3–5 times	10 (34.5)	
> 5 times	5 (17.2)	
Confidence in discussing uncertainty		20.3 ± 3.7 (10–28)
Uncertainty tolerance		15.7 ± 3.6 (7–24)
Attitude towards SDM		7.1 ± 2.8 (4–15)
Perceived social norm about SDM		9.8 ± 1.6 (6–12)

### Realism

Observers rated SPs’ behavior as equally realistic for both SPs (M = 12.4 vs. M = 12.7), which was also the case for counselors’ assessment of SPs. Counselors assessed the realism of SPs’ behavior significantly higher than observers (counselors; M = 14.9, observers; M = 12.8; t = 3.574; *p* < 0.001). Moreover, counselors assessed their consultation as moderately realistic (M = 7.8, range 5–11, with a possible maximum score of 12).

### Communicated uncertainties

#### Topics of uncertainties

Table 2 shows characteristics and quotes of counselors’ uncertainty expressions (n = 1207), organized by topic. Almost all expressions were related to scientific uncertainty (93.7%, including 42% test-related and 58% disease-related expressions) while 0.3% referred to personal and 6% to practical uncertainties. The following six topics of uncertainty were most frequently discussed, in 97% of the consultations: uncertainty regarding (1) heredity of cancer, (2) the consequences of an identified pathogenic variant, (3) the risk of developing cancer, (4) possible test results, (5) the meaning and implications of test results, and (6) possibilities of genetic techniques. Four uncertainty topics were rarely addressed by counselors: the counselees’ future in general, the cause of a pathogenic variant, whether and when to perform genetic testing, and what to communicate to counselees.

**Table 2:** The issue, topic, number of consultations, frequency per consultation, initiative and framing, and citations of uncertainties expressed by counselors during simulated consultations (n = 29; total of 1207 uncertain utterances)

Issue of uncertainty and identified topic(s) (number of consultations)	Example quote (framing)	Frequency (mean ± SD)	Counselor -initiated (n (%))	Framing	
				Directness (n (%))	Valence (n (%))
Scientific: test-related					
• Possible test results (n = 29, 100%)	*It’s the questionwhether you’ll find the causeof your disease, or whetheryou’ll find something that isn’t related or seems not to be relatedto your disease.* (direct, neutral)	10.9 ± 3.9	303 (96.2)	Direct: 286 (90.8) Indirect: 29 (9.2)	Positive: 52 (16.5) Negative: 60 (19.1) Neutral: 202 (64.1)
• The ability of genetic techniques (now and in the future) (n = 29, 100%)	*At this moment we might not knowwhat a variant means or implies, butmaybe we’ll know more in the future.* (direct, positive)	2.7 ± 1.4	77 (97.5)	Direct: 53 (67.1) Indirect: 26 (32.9)	Positive: 41 (51.9) Negative: 19 (24.1) Neutral: 19 (24.1)
• The meaning and implications of test results (n = 28, 96.6%)	*Only in a small group of people we find an unknown variant. I’ll tell you that result but will additionally tell you thatwe don’t know the associated risks. *(direct, neutral)	2.9 ± 1.7	77 (95.1)	Direct: 67 (82.7) Indirect: 14 (17.3)	Positive: 6 (7.4) Negative: 48 (59.3) Neutral: 27 (33.3)
Scientific: disease-related					
• Heredity of cancer (n = 29, 100%)	*The cancer types you have developed are not associated with one hereditary syndrome,there are multiple syndromes that could have caused them.* (indirect, neutral)	7.3 ± 3.4	207 (97.2)	Direct: 153 (71.8) Indirect: 60 (28.2)	Positive: 38 (17.8) Negative: 45 (21.1) Neutral: 130 (61.0)
• Developing cancer (with and without a pathogenic variant) (n = 29, 100%)	*Some pathogenic variantsincrease the risk of developing cancerin the long term.* (direct, neutral)	6.8 ± 3.2	192 (97.5)	Direct: 175 (88.8) Indirect: 22 (11.2)	Positive: 15 (7.6) Negative: 44 (22.3) Neutral: 138 (70.1)
• The consequences of an identified pathogenic variant (n = 29, 100%)	*An identified pathogenic variantmight imply consequencesfor relatives, depending on the pathogenic variant.* (indirect, negative)	4.6 ± 2.9	126 (94.0)	Direct: 90 (67.2) Indirect: 44 (32.8)	Positive: 19 (14.2) Negative: 48 (35.8) Neutral: 67 (50)
• Inheritance (n = 26, 89.7%)	*When you carry a pathogenic variant for cancer, we can provide relatives with certainty aboutwhether or not they carry that same pathogenic variantby testing them.* (direct, positive)	3.1 ± 1.8	76 (95.0)	Direct: 61 (76.3) Indirect: 19 (23.8)	Positive: 10 (12.5) Negative: 2 (2.5) Neutral: 68 (85)
• The presence of a pathogenic variant (n = 13, 44.8%)	*When you’ve been developing cancer or multiple cancers,you may be carrier of a hereditary predisposition* (indirect, neutral)	1.8 ± 0.9	23 (100)	Direct: 22 (95.7) Indirect: 1 (4.3)	Positive: 2 (8.7) Negative: 9 (39.1) Neutral: 12 (52.2)
• The counselee’s future in general (n = 6, 20.7%)	*You’ll never know how life goes, no one does. Youmight develop cancer in the future, maybe you’ll not.* (indirect, neutral)	1 ± 0	6 (100)	Direct: 4 (66.7) Indirect: 2 (33.3)	Positive: 1 (16.7) Negative: 2 (33.3) Neutral: 3 (50)
• The cause of a pathogenic variant (n = 3, 10.3%)	*Sometimes, due to several causes, a pathogenic variant can arise.* (indirect, neutral)	1 ± 0	3 (100)	Direct: 2 (66.7) Indirect: 1 (33.3)	Negative: 2 (66.7) Neutral: 1 (33.3)
Practical					
• Whether and when to perform genetic testing (n = 2, 6.9%)	*You should ask yourselfwhether you want to perform this test, and in particular, whether this is the right time to do so.* (direct neutral)	1 ± 0	2 (100)	Direct: 2 (100)	Negative: 1 (50) Neutral: 1 (50)
• What to communicate to patients (n = 2, 6.9%)	*Regarding uncertain findings,we should weigh whether we think it’s beneficial for you to know this informationor we think ‘we just don’t know and this information is not helpful at this moment’. *(indirect, positive)	1 ± 0	2 (100)	Direct: 2 (100)	Negative: 1 (50) Neutral: 1 (50)
Personal					
• Uncertainty discussed on meta-level (n = 26, 89.7%)	*These panels entail uncertainty. Often we can deal with this uncertainty, but of course it depends on how the person is facing it.* (direct, positive)	2.8 ± 1.7	64 (88.9)	Direct: 67 (93.1) Indirect: 5 (6.9)	Positive: 4 (5.6) Negative: 30 (41.7) Neutral: 38 (52.8)

#### Initiative and framing of uncertainty

Most expressions of uncertainty (95%) were initiated by the counselor, and framed directly (77%), e.g., *In that caseit is unknownwhat this means* vs. 23% indirectly, e.g., *It isprobably a harmless variant*. More than half of the directly-framed uncertainty expressions was furthermore framed in emotionally neutral terms (59%, e.g., *There’s a possibility to find something of which it is unknown whether it caused your cancer*) while in 26% a negative value was added, e.g., *Which might be difficult to deal with*, and in 16% of the utterances a positive value accompanied the uncertainty expression, e.g., *It might enable us to start screening*.

Ratios of framing differed between scientific test-related, scientific disease-related, practical and personal uncertainties (*p* < 0.001). Scientific test-related uncertainties were more frequently framed in direct, positive terms (15%) compared to the other topics (≤ 6%). Further, scientific disease-related uncertainties were frequently framed in indirect, neutral terms (11%).

## Counselors’ responses to uncertainty

Most of counselors’ responses to SPs’ uncertainty expressions (n = 350) were explicit (69%; i.e. referring to SPs’ uncertainty expression). Moreover, 66% of these responses reduced space for the counselee to further disclose his uncertainty. The most frequently used type of explicit space-reducing response was categorized as *information-advice*, i.e. situations where counselors would answer a question or offering reassurance by providing information (Table 3). In only 5% of their responses, counselors explicitly provided space for or explored counselees’ affective experiences with uncertainty, e.g., by addressing counselees’ ability to deal with possible uncertainty resulting from genetic testing.

**Table 3:** Number of responses, consultations, frequency and duration of responses (n = 350) during simulated consultations (n = 29)

Categories of VR-CoDES responses and their codes (number of consultations)	Number of responses (n (%))^a^	Frequency (mean ± SD)^b^
Non-explicit, reducing space (n = 24)	52 (14.9)	2.2 ± 1.1
Ignoring	20 (38.5)	1.1 ± 0.3
Shutting down	2 (3.8)	1.0 ± 0.0
Information-advise	30 (57.7)	1.9 ± 1.1
Non-explicit, providing space (n = 27)	58 (16.6)	2.1 ± 1.5
Silence	2 (3.4)	1.0 ± 0.0
Back-channel	16 (27.6)	2.0 ± 1.2
Acknowledgement	15 (25.9)	1.3 ± 0.5
Active Invitation	5 (8.6)	1.3 ± 0.5
Implicit empathy	20 (34.5)	1.5 ± 0.9
Explicit, reducing space (n = 29)	178 (50.9)	6.1 ± 2.1
Switching	11 (6.2)	1.0 ± 0.0
Post-ponement	7 (3.9)	1.2 ± 0.4
Information-advise	144 (80.9)	5.0 ± 1.9
Active blocking	16 (9.0)	1.2 ± 0.4
Explicit, providing space content (n = 21)	43 (12.3)	2.0 ± 1.1
Content acknowledgment	27 (62.8)	1.5 ± 0.7
Content exploration	16 (37.2)	1.5 ± 0.7
Explicit, providing space affective (n = 11)	19 (5.4)	1.7 ± 0.9
Affective acknowledgement	11 (57.9)	1.4 ± 0.5
Affective exploration	3 (15.8)	1.5 ± 0.7
Empathic response	5 (26.3)	1.0 ± 0.0

^a^Total number of responses for each category among the 29 consultations

^b^Mean frequency and standard deviation of responses per consultation

### Shared decision making (SDM)

Observers’ rating of counselors’ degree of SDM resulted in a mean score of 24.3 (SD = 5.6; range 6–34), which was significantly lower than counselors’ own ratings of the degree of SDM in the consultation (i.e. M = 31.4; range 22–39, respectively, *p* <  0.001).

### Associations with counselors’ characteristics

No associations between the frequency of communicated uncertainties and counselors’ level of experience, professional training, tolerance of uncertainty, confidence in communicating uncertainty, attitude towards SDM or perceived social norm about SDM was demonstrated (all r < 0.10, all *p-*values > 23). There was a trend for more experienced counselors to feel more confident about discussing uncertainty (r = 0.34; *p* = 0.08), but no association with their tolerance to uncertainty was found here (r = 0.22; *p* = 0.26), despite a trend for more confidence in discussing uncertainty being related to being more tolerant to uncertainty (r = 0.35; *p* =  0.06).

More experienced counselors were significantly *less* likely to respond to SPs’ uncertainty by providing space for further disclosure of the SPs’ uncertainty, e.g. by asking a follow-up question about the expressed uncertainty of the SP (r = 0.43, *p* = 0.02). There was a trend for counselors with a more positive attitude towards SDM to use fewer explicit responses to SPs’ expressions of uncertainty (r = − 0.33, *p* = 0.08).

Clinical geneticists scored significantly lower on degree of SDM than residents or genetic counselors (*p* < 0.03; M = 22.3 vs. M = 26.8). Also, a more negative attitude towards SDM was significantly associated with a lower degree of SDM during the consultation: r = − 0.38; *p* = 0.04. The degree of SDM was not found to be associated with years of experience (r = − 0.20; *p* = 0.29), training in communication skills (χ^2^ = 45.7; *p* = 0.32), or perceived social norm (r = 0.07; *p* = 0.72).

### Communicated uncertainties and responses in relation to SDM

A higher degree of observers’ rating of SDM was related to significantly more frequent discussion of practical and personal uncertainties (r = 0.58 (*p* < 0.01); r = 0.41 (*p* < 0.03), respectively), but not shown here to be significantly related to discussion of scientific disease-related or test-related uncertainties (r = − 0.26 (*p* = 0.17); r = − 0.08 (*p* = 0.69), respectively). No associations were found between degree of SDM and counselors’ responses towards SPs expressions of uncertainty (all r values < 0.25; all *p*-values > 0.17).

## Discussion

In this observational study using simulated patients (SPs), we aimed to gain insight into whether and how counselors discuss and address uncertainty and engage in shared decision making (SDM) when discussing a multigene panel test. Moreover, we attempted to investigate whether counselors’ characteristics were associated with their communication, responses and extent of SDM.

Nearly all uncertainties communicated by counselors pertained to scientific topics, were initiated by the counselor and framed directly, meaning that the counselor explicitly voiced uncertainty, e.g. by saying *I do not know* as opposed to *maybe*. In addition, most expressions of uncertainty were framed emotionally neutral, i.e. without a positive or negative valence. Counselors generally acknowledged SPs’ uncertainty expressions by using explicit responses; meaning that SPs’ expressions were not ignored but explicitly referred to. However, counselors’ responses mainly precluded the opportunity to first explore SPs’ uncertainties before providing information or advice. Interestingly, more experienced counselors used more space-reducing responses. Furthermore, we found a moderate level of SDM (slightly above average [27]), with clinical geneticists scoring significantly lower than other types of counselors. Consultations with relatively high SDM scores contained significantly more discussions of SPs’ practical and personal uncertainties. Our small sample size prevented us from drawing firm conclusions on other, non-significant associations with counselors’ characteristics. Not surprisingly, mainly scientific uncertainties (e.g. whether knowledge of genetic variants will increase in the future) were communicated at counselors’ initiative, since genetic counseling is primarily aimed at informing counselees about and promoting their understanding of genetic information [4, 6]. For this, counselors generally provide large amounts of information, including uncertain information [29]. More importantly, this study showed that counselors differ in the uncertain information they address: some scientific uncertainties, but particularly many practical and personal uncertainties, were not identified in all consultations (Table 2). Possibly, counselors try to enhance counselees’ understanding and recall by avoiding *information overload* and selecting the uncertain information they judge to be important to provide. Since the recall of medical information is generally low [30], this might be a sensible strategy. However, the variation in the communicated uncertainties during genetic counseling may result in practice variation causing counselees to differ in their genetic knowledge after counseling. Subsequently, counselees’ decisions regarding testing may depend more on the counselor than on the information required to be provided, e.g. as proposed in the informed consent model of Bradbury et al. [31]. Our findings indicate that consensus among counselors and genetic centers is needed regarding the topics and extent to which uncertainties should be discussed with counselees during pre-test counseling. Before we can establish such consensus, we first need to know how different approaches in communicating uncertainty affect counselees and the degree to which SDM is applied.

We also found that counselors mainly responded to SPs’ expressions of uncertainty with space-reducing responses, of which the most frequently used was *information-advice*, i.e. providing information, or offering reassurance by providing information. Counselors may use this response after an uncertainty expression of the counselee to reduce his/her uncertainty or to answer their question. However, providing information does not necessarily reduce uncertainty, as additional information may increase the complexity in understanding [5]. In addition, the content of responses may not only involve unambiguous information. A reassuring response may for example include information on the magnitude of a probability: *I understand your concern; however, the chance of developing cancer again is really small*. Hence, responding with additional information may generate ‘new’ uncertainties in counselees.

Further, results indicated that more experienced counselors used more responses that reduced space for further disclosure of uncertainty. More experienced counselors might have developed a clear strategy for the structure and content of the consultation, leaving less space and attention for other topics of uncertainty contributed by the counselee [32]. Possibly, counselors are worried that using space-providing responses may result in longer consultations. It would therefore be interesting for future research to examine whether providing more space to counselees to express their uncertainties has an impact on consultation duration and whether or not it is more efficient for counselors to address counselees’ uncertainties and to tailor the information to the individual counselee. In addition, future research should examine to what extent counselees experience (in)sufficient space to express their uncertainties. To gather such information, research involving real counselees is needed.

Regarding decision making, SDM scores are promising for the level of involvement of counselees in decision making about multigene panel testing during genetic counseling. On average, counselors’ degree of SDM was assessed as moderately, which can be considered as relatively high compared to other healthcare settings [33]. This is consistent with the findings of a recent study showing that counselors indicated to attach great value to counselees’ preferences regarding performing a panel test, as panels imply increased uncertainty [7]. Additionally, the current study showed that uncertainties were mainly framed directly and in neutral terms. This supports the principle of non-directive counseling, i.e. not necessarily raising hope or emphasizing the difficulty in dealing with uncertainty by using negative or positive terms to emphasize either one of the sides of uncertainty. A neutral framing of uncertainty allows counselees to form their own opinion about these uncertainties and to determine the weight of these uncertainties in decision-making [34]. Counselors might be aware that, regarding panel testing, there is no *best* option for an individual patient as it also involves potential drawbacks, which encourages them to avoid steering and involving counselees more extensively in decision-making [35]. However, the high number of space-reducing responses used to respond to SPs’ uncertainties raises questions about counselees’ involvement. Although no significant associations were found between the counselors’ responses to SPs expressions of uncertainty and their level of SDM, providing less space for counselees’ uncertainties may not be beneficial for their involvement in decision-making, as they are not able to share their concerns and considerations [36]. One strategy to involve counselees more extensively might be to pay more attention to exploring their uncertainties, which may also help counselors to determine what information to provide [36]. Subsequently, counselors may reduce counselees’ uncertainty by tailoring the information to their needs and uncertainties according to the tiered-binned model of informed consent [31]. This model aims to achieve informed consent by differentiating between indispensable information and information tailored to individual informational needs to support informed decision making and minimize information overload. Moreover, tailoring information will increase counselees’ ability to overthink the pros and cons of testing. Although overthinking has the potential to be detrimental for counselees, it also enables counselees to be involved in the decision about panel testing which follows ethical standards [36]. It might therefore be relevant to provide counselors with guidelines on how to optimally discuss uncertainties with counselees and involve them in decision making during consultations about panel testing. Communication skills training may be an appropriate method to equip them with the required skills for adequate counseling [37].

## Strengths and limitations

This study has some strengths and limitations that should be addressed. A first strength is that counselors of all genetic centers in the Netherlands and varying in professional training were included, providing a reasonable representation of Dutch clinical practice. Second, standardization of patient characteristics and elimination of confounding by patient characteristics was allowed by using actors instead of real counselees. This enabled us to gain insight into variation in communication behavior, as all counselors consulted with a male, 60-year old highly educated counselee. One limitation is that the number of participating counselors varied per center, possibly causing bias in the communication in clinical practice as genetic centers may differ in their approach. Second, our small sample size reduced the power to demonstrate small or even medium effects. We therefore cannot draw any strong conclusions on the non-significant associations that we found between communication and counselor characteristics. Third, using a simulated setting might have influenced counselors’ communication. Counselors perceived SPs’ behavior as realistic, however, the consultation as a whole was assessed as only moderately realistic. This may be due to our instructions to discuss a multigene panel test during an initial counseling session. As there is no standard practice in the Netherlands, centers differ in their approach to requesting and offering multigene panel testing in clinical practice. Therefore, in this study counselors may have been forced to conduct this consultation different than usual, which may have influenced their communication. Also, characteristics of the SP such as gender and educational level, may have determined counselors’ communication. Our SPs were older highly-educated men. Counselors may have perceived the SP as relatively well-literate, causing them to be relatively open about scientific uncertainty. Moreover, previous literature indicates that physicians may interact differently with male *vs*. female patients, limiting generalizability of our study findings [38]. It would be interesting for future research to assess whether counselors’ communication about uncertainty varies based on the counselee’s gender. Fourth, we used some study-specific questionnaires. For future use, our newly developed measures need to be validated. Finally, all videos were double-coded independently and, as the interrater reliability of coded uncertainties and responses was suboptimal, coded fragments were discussed in the research team to reach consensus. Identifying and coding expressions of uncertainty is, therefore, not straightforward. It would be relevant for future research to establish consensus on how uncertainty can be reliably coded.

## Conclusion and recommendations

This study provides insight into counselors’ communication of and responses to uncertainties in the context of deciding about multigene panel testing for cancer. Our findings contribute to the literature regarding counseling about multigene panel testing, which increasingly occurs in clinical practice. Our results show that cancer genetic counseling currently focuses on providing information that involves uncertainties about scientific topics. We suggest to also focus on the personal, informational needs of counselees as this might be beneficial in order to address their uncertainties and involve them more extensively in decision-making. Future research should investigate counselees’ perspective on whether/how uncertainties should be addressed. Building on insights gained from this study (and from other studies addressing the counselees’ perspective) a training for counselors in communicating about panel testing will be developed, aiming to improve their knowledge of and skills in counseling about multigene panel testing.

## Electronic supplementary material

Below is the link to the electronic supplementary material.

Electronic supplementary material 1 (DOCX 118 kb)

## References

[1] Xue Y, Ankala A, Wilcox WR, Hegde MR (2015). Solving the molecular diagnostic testing conundrum for Mendelian disorders in the era of next-generation sequencing: single-gene, gene panel, or exome/genome sequencing. Genet Sci.

[2] Hall MJ, Forman AD, Pilarski R, Wiesner G, Giri VN (2014). Gene panel testing for inherited cancer risk. J Natl Compr Cancer Netw.

[3] Howard HC, Iwarsson E (2017). Mapping uncertainty in genomics. J Risk Res.

[4] Han PK, Klein WM, Arora NK (2011). Varieties of uncertainty in health care: a conceptual taxonomy. Med Decis Mak.

[5] Han PK, Umstead KL, Bernhardt BA, Green RC, Joffe S, Koenig B, Krantz I, Waterston LB, Biesecker LG, Biesecker BB (2017). A taxonomy of medical uncertainties in clinical genome sequencing. Genet Med.

[6] Resta R, Biesecker BB, Bennett RL, Blum S, Estabrooks Hahn S, Strecker MN, Williams JL (2006). A new definition of genetic counseling: National Society of Genetic Counselors’ task force report. J Genet Couns.

[7] Medendorp NM, Hillen MA, Murugesu L, Aalfs CM, Stiggelbout AM, Smets EM (2018). Uncertainty related to multigene panel testing for cancer: a qualitative study on counsellors’ and counselees’ views. J Commun Genet.

[8] Vos J, Menko FH, Oosterwijk JC, van Asperen CJ, Stiggelbout AM, Tibben A (2013). Genetic counseling does not fulfill the counselees' need for certainty in hereditary breast/ovarian cancer families: an explorative assessment. Psycho-Oncology.

[9] Beauchamp TL, Childress JF (2001). Principles of biomedical ethics.

[10] Welkenhuysen M, Evers-Kiebooms G, d’Ydewalle G (2001). The language of uncertainty in genetic risk communication: framing and verbal versus numerical information. Patient Educ Couns.

[11] Morton TA, Rabinovich A, Marshall D, BretschneIDer P (2011). The future that may (or may not) come: how framing changes responses to uncertainty in climate change communications. Glob Environ Change.

[12] Meyerowitz BE, Chaiken S (1987). The effect of message framing on breast self-examination attitudes, intentions, and behavior. J Pers Soc Psychol.

[13] Michie S, Bron F, Bobrow M, Marteau TM (1997). Nondirectiveness in genetic counseling: an empirical study. Am J Hum Genet.

[14] Charles C, Gafni A, Whelan T (1999). Decision-making in the physician–patient encounter: revisiting the shared treatment decision-making model. Soc Sci Med.

[15] Medendorp NM, Hillen MA, Murugesu L, Aalfs CM, Stiggelbout AM, Smets EM (2018). Uncertainty in consultations about genetic testing for cancer: an explorative observational study. Patient Educ Couns.

[16] Del Piccolo L, De Haes H, Heaven C, Jansen J, Verheul W, Bensing J, Bergvik S, Deveugele M, Eide H, Fletcher I (2011). Development of the Verona coding definitions of emotional sequences to code health providers’ responses (VR-CoDES-P) to patient cues and concerns. Patient Educ Couns.

[17] O'Riordan M, Aktürk Z, Ortiz JMB, Dağdeviren N, Elwyn G, Micallef A, Murtonen M, Samuelson M, Struk P, Tayar D (2011). Dealing with uncertainty in general practice: an essential skill for the general practitioner. Qual Primary Care.

[18] Roter D, Ellington L, Erby LH, Larson S, Dudley W (2006). The genetic counseling vIDeo project (GCVP): models of practice. Am J Med Genet C.

[19] Edwards A, Elwyn G (2004). Involving patients in decision making and communicating risk: a longitudinal evaluation of doctors’ attitudes and confIDence during a randomized trial. J Eval Clin Pract.

[20] Gerrity MS, White KP, DeVellis RF, Dittus RS (1995). Physicians' reactions to uncertainty: refining the constructs and scales. Motivation Emot.

[21] Cull A, Sprangers M, Bjordal K, Aaronson N, West K, Bottomley A (2002). EORTC quality of life group translation procedure.

[22] Dormandy E, Michie S, Hooper R, Marteau TM (2005). Low uptake of prenatal screening for Down syndrome in minority ethnic groups and socially deprived groups: a reflection of women's attitudes or a failure to facilitate informed choices?. Int J Epidemiol.

[23] Ajzen I (2006). Constructing a theory of planned behavior questionnaire.

[24] Scholl I, Kriston L, Dirmaier J, Buchholz A, Härter M (2012). Development and psychometric properties of the Shared Decision Making Questionnaire–physician version (SDM-Q-Doc). Patient Educ Couns.

[25] Visser LN, Bol N, Hillen MA, Verdam MG, De Haes HC, Van Weert JC, Smets EM (2018). Studying medical communication with vIDeo vignettes: a randomized study on how variations in vIDeo-vignette introduction format and camera focus influence analogue patients’ engagement. BMC Med Res Methodol.

[26] Zimmerman PH, Bolhuis JE, Willemsen A, Meyer ES, Noldus LP (2009). The Observer XT: a tool for the integration and synchronization of multimodal signals. Behav Res Methods.

[27] Kriston L, Scholl I, Hölzel L, Simon D, Loh A, Härter M (2010). The 9-item Shared Decision Making Questionnaire (SDM-Q-9). Development and psychometric properties in a primary care sample. Patient Educ Couns.

[28] Faul F, Erdfelder E (1992). GPOWER: a priori, post-hoc, and compromise power analyses for MS-DOS [computer program].

[29] Van Zuuren F, Van Schie E, Van Baaren N (1997). Uncertainty in the information provided during genetic counseling. Patient Educ Couns.

[30] Kessels RP (2003). Patients’ memory for medical information. J R Soc Med.

[31] Bradbury AR, Patrick-Miller L, Long J, Powers J, Stopfer J, Forman A, Rybak C, Mattie K, Brandt A, Chambers R, Chung WK, Churpek J, Daly MB, Digiovanni L, Farengo-Clark D, Fetzer D, Ganschow P, Grana G, Gulden C, Hall M, Kohler L, Maxwell K, Merrill S, Montgomery S, Mueller R, Nielsen S, Olopade O, Rainey K, Seelaus C, Nathanson KL, Domchek SM (2015). Development of a tiered and binned genetic counseling model for informed consent in the era of multiplex testing for cancer susceptibility. Genet Med.

[32] Buller MK, Buller DB (1987). Physicians' communication style and patient satisfaction. J Health Soc Behav.

[33] Couët N, Desroches S, Robitaille H, Vaillancourt H, Leblanc A, Turcotte S, Elwyn G, Légaré F (2015). Assessments of the extent to which health-care providers involve patients in decision making: a systematic review of studies using the OPTION instrument. Health Expect.

[34] Karnieli-Miller O, Eisikovits Z (2009). Physician as partner or salesman? Shared decision-making in real-time encounters. Soc Sci Med.

[35] Elwyn G, Gray J, Clarke A (2000). Shared decision making and non-directiveness in genetic counselling. J Med Genet.

[36] Kunneman M, Marijnen CA, Baas-Thijssen MC, van der Linden YM, Rozema T, Muller K, Geijsen ED, Stiggelbout AM, Pieterse AH (2015). ConsIDering patient values and treatment preferences enhances patient involvement in rectal cancer treatment decision making. Radiother Oncol.

[37] Brown RF, Bylund CL (2008). Communication skills training: describing a new conceptual model. Acad Med.

[38] Hall JA, Roter DL (1998). Medical communication and gender: a summary of research. J Gend&nbsp; Specif Med.

