# Supplementary material for: ‘We don’t know for sure’: discussion of uncertainty concerning multigene panel testing during initial cancer genetic consultations
Source: Fam Cancer. 2019 Nov 26;19(1):65–76. doi: 10.1007/s10689-019-00154-4 (PMC7026220; doi:10.1007/s10689-019-00154-4)
Supplement: Supplementary file 1 — Electronic supplementary material 1 (DOCX 118 kb) [file 10689_2019_154_MOESM1_ESM.docx]

**Supplement A**

**SPs’ script**

*Background*

Clinical genetics is a discipline that is evolving rapidly. One example are multigene panel tests which are usually performed to find out if someone is carrying a genetic predisposition for cancer. Nowadays, these panel tests are increasingly performed. A panel test involves sequencing multiple genes simultaneously to identify deviations (often called mutations). The number of genes tested in a panel varies, from a few till hundred.

Besides many advantages, such as an increased probability to identify the cause of cancer, there are also some disadvantages. One example is that variants can be identified of which the meaning and implications are less well-known. This leads to uncertainty, for example, uncertainty about the consequences of a mutation for the patient. Studies have shown that communicating uncertainty has the potential to decrease the level of satisfaction and increase the level of anxiety in patients. In addition, uncertainty could also lead to patients’ avoidance making decisions. Often, during genetic counseling, decisions whether or not to perform a panel test are made. As panel test may comprise uncertainties for patients afterwards, it is desired that patients in whom a panel may be performed, are informed and involved in deciding about performing such a panel.

You may think of *shared decision making* in these consultations. In summary, shared decision making could be explained as 'the patient and physician make a decision together based on the best available information. As a 'partner' of the doctor, the patient is encouraged to consider all possible options, as well as the advantages and disadvantages of these options, communicate their preferences and receive help to select the option that is best suited to their preference(s).

Due to the increased level of uncertainty concerning panel testing, it is desired to communicate uncertainty to enable shared decision making during genetic counseling. The current study provides insight into whether and how uncertainty concerning panel tests for cancer is communicated in clinical practice.

*Aim of this study*

The aim of this study is to gain insight into whether and how counselors communicate uncertainty concerning panel tests for cancer and whether the communication is associated to their characteristics. The results of this study show insight into current communication of uncertainty, and will be used for future research to investigate the effects of different ways of communicating uncertainty and inform the development of communication training for counselor.

*Case*

You are Bas de Graaff, a 56 years old man. Today, you are visiting for genetic counseling alone because your wife passed away due to a car accident four years ago, and your children live far away have to work today. Your family has been through a difficult time due to the death of your wife, and the fact that you have gotten cancer three times. However, all of this has made your family stronger, and you can share your feelings and support each other. You do not have professional help to deal with it because you can handle it together with the support you receive from your family and friends. Your relationship with your children is very good and from time to time you look after your granddaughter. Your children offered to come along to the counseling session, but you didn't want to burden them again as they already need to assist you during the chemotherapy you receive for the treatment of your colon cancer.

You are employed and have no future plans to start your own business. Normally, you work four days a week but since you receive chemotherapy for colon cancer, you are called in sick.

If the genetic counselor mentions possible consequences for insurance; you react slightly surprised and ask for the exact consequences. (clarification: the indication of a genetic predisposition could affect the insurances you need to take when you become self-employed).

When you were 40 years old, you were diagnosed with renal cell carcinoma and when you were 51 years old you were diagnosed with prostate cancer. You were so happy to be done with it, until recently you visited your general practitioner because of heavy abdominal complaints and you were diagnosed with colon cancer. The immunohistochemistry assessment that was done on the tumor did not show any abnormal results. You do know about this assessment, yet you cannot remember the name but do remember that it appeared that the tumor is not genetically determined. Having cancer for the third time has a serious impact on you, and you are worried about the reason why you have developed it again, but at the same time you are very strong: 'you don’t give up’.

Before being diagnosed with renal cancer you were healthy and still you don’t have health problems besides the cancer. Some of your relatives have also been diagnosed with cancer in the past, like your sister who has been diagnosed with breast cancer. Fortunately she is fully recovered. The pedigree on the next page shows your complete family history. Your family is non-Jewish (clarification: this has been associated with an increased risk of developing breast cancer). If the genetic counselor talks about a letter to inform your family, you react slightly surprised. You have been thinking about your children but you did not consider telling the rest of the family. Informing your relatives should however not be a problem, you don't see them very often but are still in contact with them (you were however not informed on forehand about the possibility that you might have to inform your relatives). If the counselor asks about how you are planning to inform the family, you will tell him that you would call them, or tell them or give them the family letter whenever you will see them in the near future.

During the consultation you are going to have with the genetic counselor, a genetic panel test to find out whether you carry a genetic predisposition for cancer is going to be discussed.

**Note:** When playing the patient you have no prior knowledge about panel tests and you do not know this is going to be discussed during counseling. The information provided above is only provided to clarify the consultation and your role as a patient.

Family history

The counselor will probably start discussing your pedigree with the help of the information you have sent to him/her prior to this consultation. Subsequently, the counselor will inform you about the genetic test and the procedure concerning disclosing the test results. Keep the medical information showed below in mind. It is not necessary for you to bring up the information, yet it can be helpful in answering questions of the counselor. When something is asked that is not provided in the information below, please come up with an answer yourself and try to answer neutrally.

**Note:** It is possible that a counselor follows a different approach regarding the consultation. This should not affect you, let the counselor lead you through the conversation.

Medical information

- At the moment you are suffering from colon cancer
- You has surgery for your colon cancer and in addition, your currently receive chemotherapy
- At the age of 40, you have had renal cancer. One kidney was affected: this one is removed and you needed to take medication afterwards.
- At the age of 51, you have had prostate cancer. You received radiation treatment and currently your prostate cancer is under control.
- You have no other health problems.
- The tumor in your colon did not appear to be genetically determined. This is tested with a coloring test on the tumor.
- Polyps have never been found in your colon; you do not experience problems with your lungs; you don’t have any lumps on your tongue; there are no dimples on your hands; no cysts in your jaw or at any other body parts.
- You are not well informed about health problems in your family like benign tumors.
- You are almost certain that your sister is not genetically tested, otherwise, she would probably have told you. For other relatives, you are not up to date.

Personal information

- You are 56 years old
- You are a widower
- You have three children, two daughters (28 and 26 years old) and a son (25 years old).
- You have a granddaughter from your oldest daughter (6 months old)
- You don't smoke
- You drink a maximum of 5 to 6 glasses of alcohol at weekends

Your family is non-Jewish

- You have a good relationship with your family, not really close, but you would be able to call them
- You told your children about this counseling appointment and they fully support you. You did not discuss yet whether your children would like to get tested as well. You think they might be positive about testing but you are not certain. No other relatives know about this appointment
- You think no one on your mother's side of the family has had cancer
- Your father died of old age, not due to his prostate cancer

Additional

- Mr. de Graaff’s expectation of this counseling session: to gather more information about inheritance of cancer as he already developed cancer multiple times. He thinks it is particularly important because of his children and granddaughter.
- The reason for counseling: His oncologist has referred him to the clinical genetics department as he taught it would be a good idea. Mr. de Graaff agrees with that because he would really like to know why he has developed cancer three times.
- When the counselor asks: Did someone already told you about clinical genetics or inheritance? Answer: No, barely, only that I could possibly receive information about inheritance here

*Pedigree*


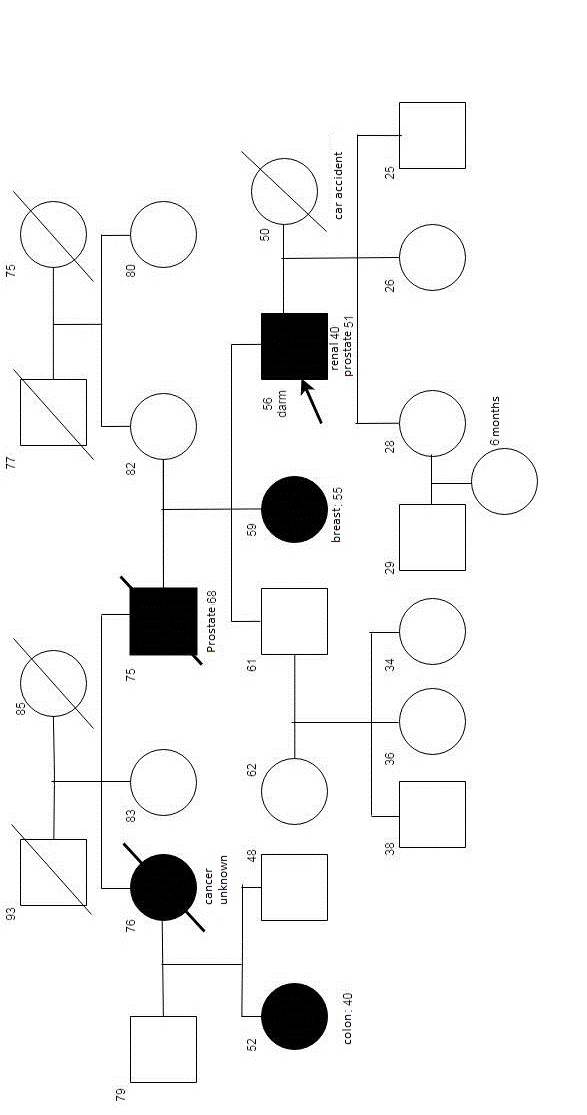


- **The arrow** refers to you (Mr. de Graaff)
- **Circle:** Female
- **Square**: Male
- **Black**: Person is affected by a form of cancer (type of cancer + age are shown below)
- **Strikethrough**: a person has passed
- Other numbers refer to age

*General instructions*

Standard behavior

You are a patient and you are not aware of what is written above. It is especially important for this study that you continuously play the “same patient”. This means that the patient should not be very quiet towards one counselor and very talkative towards another counselor. Be consistent in interaction with the counselor and with giving information. Yet, try to respond to every question the counselor is asking and it is ok to interrupt the counselor to ask a question yourself.

Average and realistic

It is important to play a realistic, average patient. Do not be too overbearing, too rational or too emotional. You can express your concerns about your health, the health of your (grand) children and about the fact that it is possible that you carry a mutation and what consequences that might have for you.

Reason of visit

If the counselor asks about the reason for visiting him/her, you respond by saying you were referred by your oncologist. This is a relief for you because you would like to find out why you have developed cancer three times; you want to receive certainty. Besides, you would like to have certainty for your children and granddaughter, so you could protect them from developing cancer.

Uncertainty from the counselor

The focus of this consultation is the communication about multigene panel tests during genetic counseling. In particular: the *manner* in which counselors communicate uncertainty. We expect that counselors communicate uncertain information about the panel test. There different forms of uncertain information that you could expected to be communicated:

- Risk information, like the risk of having cancer or carrying a mutation.
- Uncertainty about the ability to detect mutations, like ‘When no mutation is found in the test it does not necessarily mean that it is not hereditary’.
- Uncertainty about the meaning of a mutation, like: ‘When performing this test, we could identify variants/ mutations/deviations of which it is unknown what it means.
- Uncertainty about the consequences of a mutation, like: ‘We could identify variants or mutations of which the consequences are unknown’.
- Uncertainty about screening, like: ‘We could identify something of which we cannot offer any screening’.
- Other uncertainty expressions, like: ‘I could not predict how this will end’.

If the counselor shares this kind of uncertain information with you, ask about what it means for you/ what the counselor means by that/ what it could mean for your (grand)children. If the counselor does not provide any space for questions, respond in the way you would usually do.

Uncertainty from the simulated patient

Besides responding to the counselor, we need you to express some uncertainties as well. What you should express **at least,** is:

1. ‘I’m visiting because I want to receive certainty about a genetic predisposition/ I would like to have certainty. A variation could be: ‘So this test does not necessarily provide certainty, and I am here to receive certainty, so why would I choose to perform this test?’ You could express this after the genetic counselor has briefly provided any information on panel tests.
2. ‘Oh, what if something unknown is determined... What am I supposed to do then..’
3. ‘What if I develop cancer again, I really don’t want that, I have already had it so often.’

Depending on the space that the counselor provides you to ask questions, you could ask some questions. For example, pick one out of these following categories (or of both):

1). – How does this DNA test work?/ What will I need to do for this DNA test?/ Where could I go to give blood for the DNA test?

- How long will it take before the test results are available? / How will I receive the test result?

2). - What will an unknown variant mean for my future?

- What would an unknown variant mean for my children and grandchildren?

You are also free to ask other questions that you come up with during the conversation! The more natural it looks, the better, so don’t hesitate!

It would be nice to at least ask the doctor how long it would take to receive the results; this provides insight into the variation!

Decision

We expect that the counselor 1) leaves the decision up to you, 2) advises you to perform the test or not, 3) encourages you to postpone the decision (‘you should think about it’)

1. If the counselor asks you explicitly about your preferences whether or not to perform the test; respond by saying you want to perform it. If the counselor says ‘what would you like to do?'; respond by saying: ‘I really would like to know why I have had cancer three times and my children can be protected, so I am leaning towards taking the test to find an answer’.
2. If the counselor explicitly advises you to choose to perform the panel test or not, follow his/her lead.
3. During this consultation, it is not necessary to make a decision. Yet do not steer towards the option of ‘thinking about a bit more' yourself, wait until the counselor says this and then go along with it. If the counselor encourages the ‘thinking about it a bit more’, by saying, for example, to discuss it with your children, respond slightly surprised: of course, this is an option as well and you like this idea.

If in any case, a different decisions-scenario occurs, let the counselor take the lead and follow him/her.

Your blood it already taken often for the chemo, so when the counselor suggests this, it would be a good moment to take some blood for this genetic test as well. You would prefer to visit again to receive the test results.

General recommendations

- Do not ask explicitly: ‘What would u do’, ‘What would u advice?’ or ‘What do most people do’
- Let the counselor take the lead. It is not necessary that you provide all information written in this case. However, do share your questions, concerns, and wishes with the counselor.
- Ask for clarification when unstructured or unclear information is given. Also when too much information is given at once. Do this according to what feels right to you.

*Mindset during counseling*

You will enter the conversation with an open mind. You didn’t realize that such expanded genetic tests exist, but you are curious about them.

You are really focused on your treatment for colon cancer. Besides that, you worry about the fact that this is the third time you have developed cancer, and that some relatives have also dealt with cancer. Is that an indication that it is inherited? Your concerns may be well expressed during this consultation. You want to know the reason for the cancers at all costs, especially for your children and granddaughter. You are very much concerned about their health and you really don't want them to develop cancer. If these tests could of any help in this, you would really want to choose for it.

**Note:** whenever the counselor offers to provide you with any information (‘Do you want to know more about…? / I could tell you about…?’), always respond by saying that you would like that.

*Finally*

Questionnaire counselors

Avoid giving feedback directly after the consultation. Directly afterwards, the counselors will complete a questionnaire assessing their satisfaction with the consultation and their perception of the degree of shared decision making. Therefore it is important that you don’t provide any feedback during and immediately after the consultation. After the counselor has completed the questionnaire, it is allowed to do so.

*Additional advice*

- When the counselor asks specific questions about your relatives, respond by saying that you don’t know this.
- Don’t go to deep into the uncertainty about the fact that it is not guaranteed that it is not hereditary when nothing is found, as the current genetic techniques are not able not provide this certainty yet. ‘Real life patients’ usually don’t, so respond like you think this is understandable. Yet, do respond to the possibility of receiving uncertain test results.
- Do not play a very wise patient. Responses or remarks like ‘an unknown variant actually means that you receive too much information' are very wise, so avoid this.
- Don’t use the words uncertain or uncertainty too much, only to express that you visit and want to perform the test to receive certainty. But don’t use these words in other sentences.

**Supplement B**

**Development and content of the coding scheme**

Based on literature [4] and our own recent qualitative work on uncertainty communication in cancer genetic counseling [7, 15], we first developed a basic coding scheme. After discussion with the full research team, small adaptations and additions were made to this coding scheme. Two trained researchers (NM and PvM) next independently coded the four pilot videos. After each video, codes and coded fragments were discussed and the coding scheme was adjusted. After consensus was reached, the final coding scheme was discussed among and approved by the full research team.

Regarding expressions of uncertainty, the final coding scheme included 41 detailed codes covering 12 topics about which uncertainty could be expressed: (1) possible test results (e.g., uncertainty about which test result to expect or to be prepared for); (2) the present and future possibilities of genetic techniques (e.g., uncertainty about future possibilities to test for genetic predispositions); (3) the meaning and implications of test results; (4) inheritance; (5) the presence of a pathogenic variant; (6) heredity of cancer (e.g., uncertainty about whether the counselee’s cancer is hereditary); (7) the consequences of an identified pathogenic variant; (8) developing cancer (with and without a pathogenic variant); (9) the counselee’s future in general (e.g., uncertainty about what to expect in the future); (10) the cause of a pathogenic variant (e.g., how the counselee obtained this pathogenic variant); (11) whether and when to perform testing; and (12) what and when to communicate to patients (during the current consultation and after testing, regarding the test result). During subsequent coding of study videos, one additional topic was identified: uncertainty on a meta-level. This topic differed from the others as it involved uncertainty on a more abstract level, e.g. a counselor mentioning the existence of uncertainty related to panel testing, instead of the concrete expression of uncertainty about a certain topic. This topic was included in the coding scheme, resulting in a total of 13 topics. In Box 1 the classification of codes among their topic and overarching issue (according to the framework of Han and colleagues [4]) are shown.

**Box 1.** Classification of codes of counselors’ utterances of uncertainty and their topic and overarching issue.

| Scientific: test-related   1. Possible test results  - probability of (not) identifying a pathogenic variant (for cancer) - probability of and/or uncertainty about (possible) test results - probability of secondary finding - probability of uncertain secondary finding - uncertainty about secondary finding - uncertainty about uncertain secondary finding - probability of pathogenic variant/variant in unknown gene - probability of variant of unknown significance (VUS)  1. The ability of genetic techniques (now and in the future)  - uncertainty about scientific possibilities (now) - uncertainty about scientific possibilities (in the future)  1. The meaning and implications of test results  - uncertainty about the meaning of a pathogenic variant - uncertainty about the meaning of an secondary finding   Scientific: disease-related   1. Inheritance  - Probability of and/or uncertainty about inheritance of a pathogenic variant  1. The presence of a pathogenic variant  - Probability of and/or uncertainty about (carrying) a pathogenic variant  1. Heredity of cancer  - probability of hereditary cancer - uncertainty about the cause of cancer - uncertainty about hereditary - uncertainty about gene-cancer association  1. The consequences of an identified pathogenic variant  - Uncertainty about familial consequences - Uncertainty about consequences for patient - Uncertainty about communicating to family - Uncertainty about screening (in general)  1. Developing cancer (with and without a pathogenic variant)  - probability of and/or uncertainty about developing cancer - probability of and/or uncertainty about recurrence of cancer - probability of and/or uncertainty about developing cancer when carrying a pathogenic variant  1. The counselee’s future in general  - uncertainty about the future - counselors’ uncertainty to make predictions  1. The cause of a pathogenic variant  - uncertainty about the cause of a pathogenic variant   Practical   1. Whether and when to perform genetic testing  - uncertainty about when to perform testing - uncertainty about whether to perform  1. What to communicate to patients  - uncertainty about what to communicate to patients - uncertainty about when to communicate to patients   Personal   1. Uncertainty discussed on meta-level  - uncertainty on meta level |
| --- |

To code counselors’ responses to SPs’ utterances of uncertainty, the Verona Coding Definitions of Emotional Sequences (VR-CoDES-P) were used [16]. The VR-CoDES system is an international descriptive coding system for patients’ expressions of cues or concerns and the subsequent response of the physician. In this study, the VR-CoDES were used to code counselors’ responses to SPs’ concern, i.e. scripted and spontaneous utterances of uncertainty in terms of their explicitness (whether or not a clear reference was made to the counselee’s uncertainty), and whether counselors *reduced* or *provided* *(affective or content)* space by inviting or allowing the SP for further disclosure of the uncertainty. The combination of explicit/non-explicit and space-providing or reducing responses led to five response categories: 1) non-explicit, reducing space (NR); 2) non-explicit, providing space (NP); 3) explicit, reducing space (ER); 4) explicit, providing space *content* (EPC); and 5) explicit, providing space *affective* (EPA).

These five response categories each comprise multiple possible responses (Fig. 1). NR responses include ignoring (e.g., no reference whatsoever to uncertainty), shutting down (e.g., ‘*Let’s move on’*) and information advise. NP responses include silence, back channel (stimulating by using ‘*Mmm*’ or ‘*Ok*’), acknowledge (e.g., non-specifically acknowledging the content: ‘*I see*’), active invitation (e.g., a question without referring to uncertainty), and implicit empathy (e.g., an empathic response: ‘*I understand’*). ER responses include switching (e.g., asking about related symptoms), post-ponement (e.g., ‘*I’ll like to talk about your feelings in a minute*’), information advise, and active blocking (‘*Don’t worry!*’). EPC responses include acknowledgement (e.g., referring to the content of uncertainty: counselee: ‘*I worry’,* counselor*: ‘..about passing on the pathogenic variant’*), and exploration (e.g., asking about the content of uncertainty). Finally, EPA responses include acknowledgement (e.g., referring to the emotional aspect: ‘*Worry..*’), exploration (e.g., asking about the emotional aspect), and empathy (e.g., legitimizing or sharing the counselee’s uncertainty).

**Supplement C**

**Verona Coding Definitions of Emotional sequences**


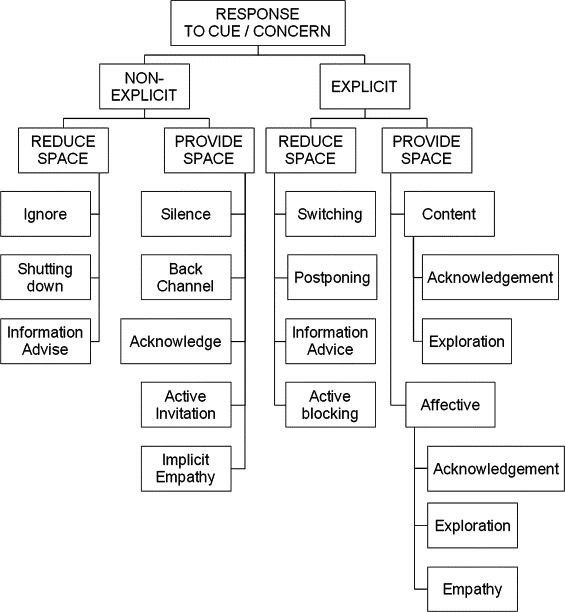


**Fig. 1.** The VR-CoDES flow chart.
